# Hyperpolarised magnetic resonance for in vivo real-time metabolic imaging

**DOI:** 10.1136/heartjnl-2017-312356

**Published:** 2018-04-27

**Authors:** Andrew Apps, Justin Lau, Mark Peterzan, Stefan Neubauer, Damian Tyler, Oliver Rider

**Affiliations:** 1 Oxford Centre for Clinical Magnetic Resonance Research (OCMR), Division of Cardiovascular Medicine, Radcliffe Department of Medicine, University of Oxford, Oxford, UK; 2 Department of Physiology, Anatomy and Genetics, University of Oxford, Oxford, UK

**Keywords:** cardiac magnetic resonance (CMR) imaging, coronary artery disease

## Abstract

Although non-invasive perfusion and viability imaging often provide the gateway to coronary revascularisation, current non-invasive imaging methods only report the surrogate markers of inducible hypoperfusion and presence or absence of myocardial scar, rather than actually visualising areas of ischaemia and/or viable myocardium. This may lead to suboptimal revascularisation decisions. Normally respiring (viable) cardiomyocytes convert pyruvate to acetyl-CoA and CO_2_/bicarbonate (via pyruvate dehydrogenase), but under ischaemic conditions characteristically shift this conversion to lactate (by lactate dehydrogenase). Imaging pyruvate metabolism thus has the potential to improve upon current imaging techniques. Using the novel hyperpolarisation technique of dynamic nuclear polarisation (DNP), the magnetic resonance signal of injected [1-^13^C]pyruvate can be transiently magnified >10 000 times over that seen in conventional MR spectroscopy, allowing the characteristic metabolic signatures of ischaemia (lactate production) and viability (CO_2_/bicarbonate production) to be directly imaged. As such DNP imaging of the downstream metabolism of [1-^13^C]pyruvate could surpass the diagnostic capabilities of contemporary ischaemia and viability testing. Here we review the technique, and with brief reference to the salient biochemistry, discuss its potential applications within cardiology. These include ischaemia and viability testing, and further characterisation of the altered metabolism seen at different stages during the natural history of heart failure.

## Metabolic imaging: the prospect of overcoming fundamental limitations of contemporary cardiovascular non-invasive imaging

The clinical impact of revascularisation procedures, whether treating stable coronary artery disease, or restoring function to hibernating myocardium, is highly variable. Prognostic impact is not definitively demonstrated,^1 2^ and in the face of contemporary medical therapy, even antianginal effects may be minimal.^3 4^ Ischaemic burden confers risk,^5^ yet despite iterations of functional testing purporting to measure just this, procedural benefit is still only restricted to reducing future urgent revascularisation events with no reduction in myocardial infarction, death or increase in exercise capacity.^3 6^

In vivo metabolic imaging of the downstream metabolism of [1–^13^C]pyruvate with dynamic nuclear polarisation (DNP) has the power to directly identify the hallmarks of both ischaemia and viability by imaging the switch from aerobic to anaerobic metabolism in ischaemia (pyruvate conversion to lactate) and aerobic respiration itself for viability (bicarbonate production). In contrast, contemporary techniques only report surrogate markers of ischaemia (coronary flow or myocardial perfusion) or in the case of viability the inference of live tissue from the measurement of dead tissue.^7 8^ Hyperpolarised metabolic imaging with DNP potentially adds sensitivity and specificity to inform the targeting of revascularisation procedures and diagnosing ischaemia itself. Such ‘metabolic phenotyping’ has implications beyond coronary artery disease. DNP offers the opportunity to extend our knowledge of energy homeostasis and substrate handling in heart failure, such that subtle metabolic shifts preceding ventricular decompensation in at-risk groups can be appreciated. This potentially lends opportunity for more timely interventions than afforded by conventional serial structural imaging alone. In this review, we appraise this new technology, which may become a simple, quick adjunct to contrast-enhanced stress perfusion and viability MR protocols and may in the future potentially even replace those. With reference to the underlying biochemistry, and to published preclinical data which alludes to the potential of its cardiac clinical translation, we review its candidate cardiovascular applications. A brief summary of these are shown in table 1.

**Table 1:** Potential cardiac applications of hyperpolarised magnetic resonance technology

Potential cardiac application	Biochemical basis	Strengths	Weaknesses	Alternative approaches
Ischaemia testing in coronary artery disease	Hypoxia curtails Krebs cycle flux. Pyruvate metabolism switches from PDH to LDH—resulting [1- ^13^C]lactate can be imaged.	By imaging [1-^13^C]lactate, the technique can measure the biochemical hallmark of ischaemia, not a surrogate marker.	Sensitivity for low-grade ischaemia is yet to be seen; the models studied are reperfusion models. Extra – cardiac [1-^13^C]lactate makes cardiac imaging difficult.	Conventional functional ischaemia testing: (1) non-invasive: stress perfusion CMR, SPECT– MPI* and DSE; and (2) invasive: FFR and iFR†
Viability testing in coronary artery disease	Viable myocardium must be respiring with flux through PDH. The CO_2_ produced equilibrates with HCO_3_^−^. H^13^CO_3_^−^ production therefore defines viable myocardium.	Unlike LGE, detection of H^13^CO_3_^−^ delineates alive tissue with potential for recovery, potentially refining the group revascularised.	Resolution – human cardiac imaging down to 8.8 × 8.8 × 10 mm is demonstrated. Reasonable SNR at higher resolution requires further technical development. This compares with 1.4 × 1.6 × 5 mm resolution for LGE.	Conventional testing: (1) CMR – LGE imaging and contractile reserve. (2) SPECT – perfusion and contractile reserve. (3) DSE – contractile reserve.
Heart failure (general considerations)	Energy substrate handling changes with stepwise progression towards heart failure, initially with increased glucose usage. PDH in part regulates the balance between fatty acid and glucose metabolism.	Serial ^13^C imaging and metabolic phenotyping of energy substrates in those at risk of developing heart failure may inform risk stratification and treatment regimens.	Identification of shifts in substrate handling may simply reflect progression of heart failure and may not represent an opportunity for an intervention that alters progression.	None comparable. Contemporary strategies of serial structural imaging with various modalities simply reports function only.
Heart failure (defining the aetiology)	Insulin resistance and raised circulating fatty acids result in markedly reduced glucose oxidative ability in diabetic cardiomyopathy. Metabolic phenotyping may inform diagnosis in the failing heart.	Characterising the metabolic hallmarks of cardiomyopathy may aid diagnosis in circumstances such as unexplained hypertrophy (HCM vs hypertensive vs storage disorders), and identify those eligible for targeted metabolic therapy	As for heart failure – general considerations.	As yet metabolic phenotyping has not yet reached the clinic in this circumstance, however, ^13^C imaging would be a helpful adjunct to current imaging modalities.
DNP – the technique, compared with other metabolic imaging modalities	The magnetic energy (polarisation) and hence MR signal of the ^13^C tracer is increased up to 10 000 times. Achieved by transferring the high polarisation associated with free electrons at very low temperature by microwave irradiation.	Allows in vivo real-time imaging of normal and abnormal metabolism, and the study of how this contributes to the disease phenotype.	(1) Technically challenging: dissolution from 1K, pH neutralisation and radical removal must happen extremely fast for signal. (2) Only fast metabolic reactions can be studied. (3) Technical advances in coils and sequences needed to improve imaging resolution. (4) Imaging fundamentally relies on tracer delivery.	Other metabolic imaging: 1) PET: only able to measure uptake of a radioactive tracer (eg, ^18^F– FDG) not its metabolic fate. 2) MRS: mostly limited to ex-vivo hearts due to the need for enriched ^13^C substrates and extended imaging times.

*Single photon emission computed tomography – myocardial perfusion imaging.

†Instantaneous wave free ratio – hyperaemia free intracoronary functional assessment.

CMR - Cardiovascular Magnetic Resonance; DNP, dynamic nuclear polarisation; DSE, Dobutamine stress echocardiography; FFR, Fractional flow reserve; iFR, instantaneous wave-free ratio - hyperaemia free intra-coronary functional assessment; LDH, lactate dehydrogenase; LGE, late gadolinium enhancement; MRS, Magnetic resonance spectroscopy; PDH, pyruvate dehydrogenase; PET, Positron emission tomography; SPECT - MPI - Single photon emission computed tomography.

## Metabolically defining ischaemia and hibernation with the fate of pyruvate: *the* crossroad metabolite

**Figure 1:**
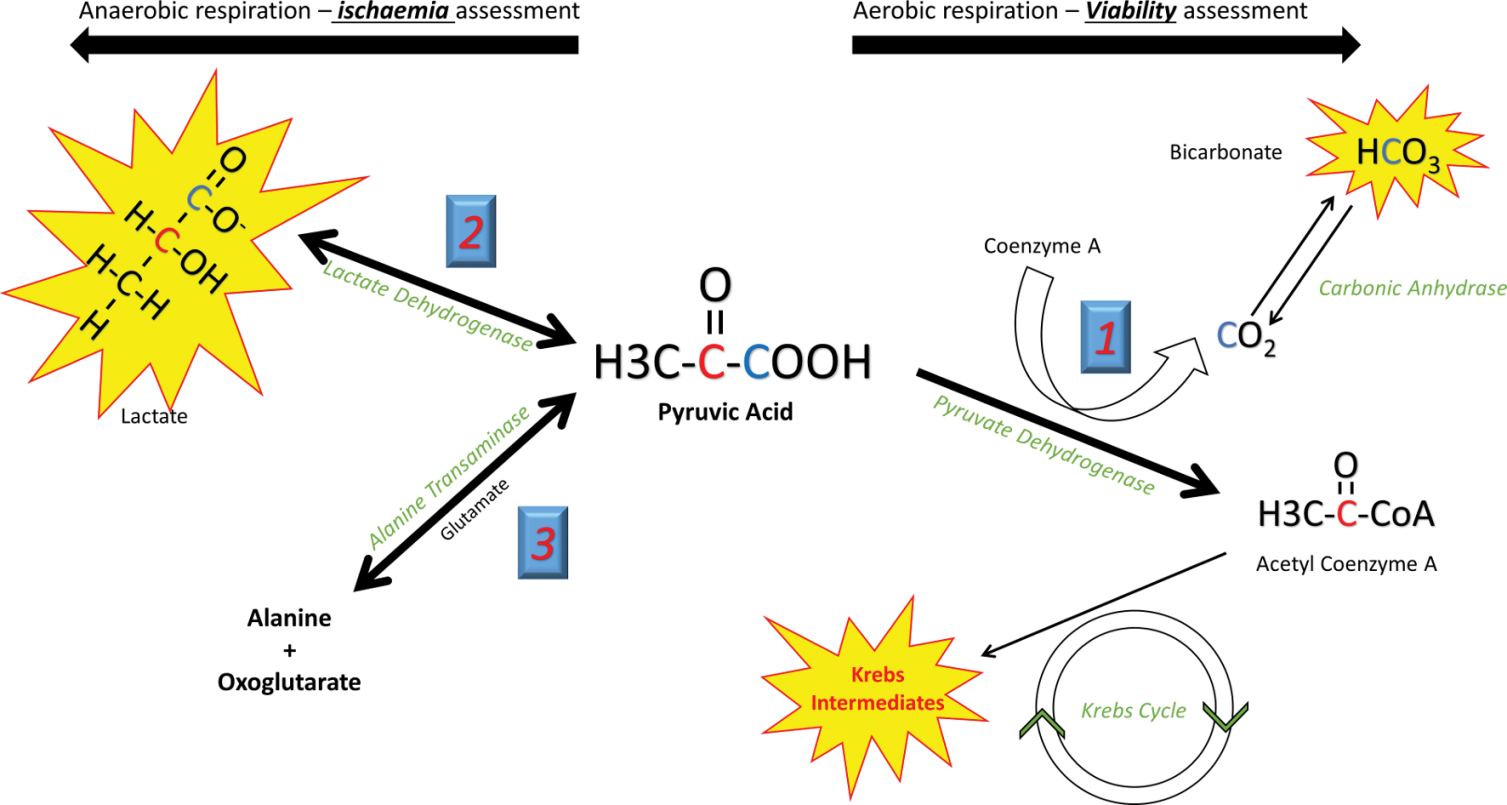
Different metabolic reactions can be studied according to the position of the ^13^C label in pyruvate. In the C1 position (blue), during aerobic respiration HCO_3_ offers a measure of flux through PDH and hence aerobic respiration. Its production can thus be used to define viable (living) myocardium. In ischaemic conditions lactate production is seen. In the C2 position (red) the ^13^C label is passed directly to acetyl-CoA and into the Krebs cycle. Krebs cycle flux can be measured via the spectra of the intermediate metabolites. PDH, pyruvate dehydrogenase.

Pyruvate, the end product of glycolytic glucose breakdown, sits at a crossroads of key metabolic pathways. A brief discussion of these is needed to appreciate its power as a metabolic tracer. The fate of pyruvate is determined by the prevailing metabolic condition, impacted by both the cellular capacity for aerobic respiration via oxidative phosphorylation, and the relative contribution of carbohydrates and fatty acids as the substrate for energy production. Three competing metabolic conversions of pyruvate must be considered (figure 1). Pyruvate dehydrogenase (PDH) within the mitochondria irreversibly catalyses the oxidative decarboxylation of pyruvate to CO_2_ and acetyl-CoA (a two-carbon unit that enters the Krebs cycle producing Reduced nicotinamide  adenine dinucleotide  (NADH)/Reduced flavin adenine dinucleotide (FADH_2)_ to drive oxidative phosphorylation and substrate level phosphorylation of Adenosine Triphosphate  (ATP)). During hypoxia, energy requirements exceed the capacity of oxidative phosphorylation and Krebs cycle flux becomes limited due to l bioavailability of NAD^+^/FAD. Here pyruvate is instead converted to lactate (a freely reversible reaction catalysed by lactate dehydrogenase (LDH)), regenerating cytosolic NAD^+^ and allowing an increased glycolytic contribution to ATP production in the absence of oxygen. Glucose becomes the preferential energy substrate, and glycolytic flux is increased.^9^

By determining the fate of a [1–^13^C]-labelled pyruvate substrate, hyperpolarised metabolic imaging has the potential to directly identify ischaemic myocardium with LDH transferring the ^13^C label to lactate. Similarly, direct identification of the metabolic hallmark of viability, continued cellular respiration, is possible by assessing PDH flux. The ^13^C label here is transferred to ^13^CO_2_, which rapidly equilibrates with H^13^CO_3_^−^ via carbonic anhydrase and can be detected. When in the second carbon position, the ^13^C nucleus is transferred directly from [2–^13^C]pyruvate to acetyl-CoA by PDH, and hence can be tracked through Krebs cycle intermediates, giving a direct measurement of Krebs cycle flux. PDH regulation determines the relative contributions of glucose and fatty acid oxidation to the heart’s energy requirements. During health, beta oxidation of long-chain fatty acids is the primary contributor to the acetyl-CoA pool, with that derived from glucose (via glycolysis) becoming predominant during adrenergic stress,^10^ ischaemia^11^ and during the later stages of heart failure.^12^ Hyperpolarised imaging can also quantify these cellular switches due to the associated alterations in PDH flux in various pathological states.

## The evolution of metabolic cardiac imaging and the road to hyperpolarised MRI

**Figure 2:**
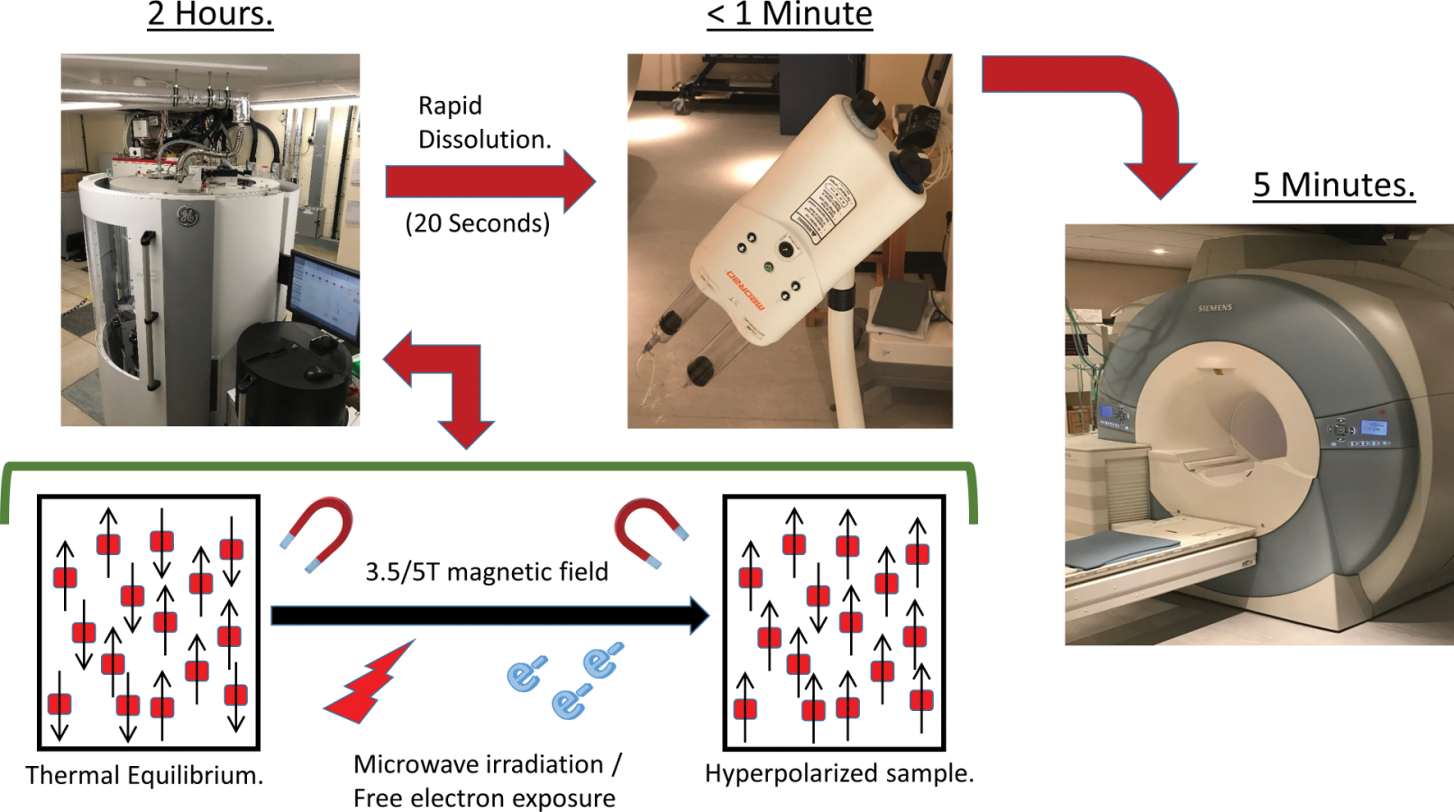
Dynamic nuclear hyperpolarisation theory: in a magnetic field, nuclei behave like bar magnets aligning with or against the applied field. The tiny difference in these two populations at thermal equilibrium produces a small MR signal (polarisation of 0.0005%). DNP magnifies the difference in these two populations and hence the MR signal >10 000 fold. This is achieved by mixing the ^13^C containing nuclei with a source of free electrons (a radical) at very low temperature (~1 Kelvin). At these low temperatures, free radical polarisation reaches 100%, and microwaves are used to transfer this electron polarisation to the nuclear spins. Dissolution is rapid to ensure the signal is maintained long enough to allow injection into the patient and in vivo metabolism to be observed.

Clinical grade hyperpolarised metabolic imaging is in its infancy in humans but is here. With its ability to detect compounds containing the non-radioactive carbon-13 (^13^C) nuclei, magnetic resonance spectroscopy (MRS) is ideally suited to studying cardiac metabolism and has the advantage of being radiation free, with the power to track downstream metabolites. However, the technique is inherently limited by sensitivity. Conventional MRI relies on the abundance of hydrogen nuclei (protons) in water to produce adequate signal. ^13^C MRS is limited by the low magnetic energy (polarisation) of the nuclear spins compounded by the very low abundance of the ^13^C isotope,^13^ with most experiments limited to ex vivo hearts perfused with concentrated ^13^C-labelled substrates, and imaging undertaken over several hours to follow the downstream metabolic fate. The technique of hyperpolarisation, via DNP, allows amplification of the MR signal from the ^13^C-infused substrate by more than 10 000 times by enhancing the polarisation of nuclear spins, rendering real-time metabolic imaging feasible.^14^ This is achieved by transferring the high electron spin polarisation associated with free electrons at low temperature to the nuclear spins of the sample.^15^ The tracer is mixed with a free radical to provide the source of free electrons, then frozen in liquid helium at a temperature of ~1K within a high magnetic field strength; under such conditions, the polarisation of the free electrons approaches 100%. This uniform polarisation is then transferred to the nuclear spins of the sample by irradiation with microwave energy, the frequency being determined by both the magnetic field strength and tracer used. The process takes in the order of 60 min, and results in ^13^C polarisation of up to 50%, far higher than that encountered in normal MRI (0.0005%) (figure 2).

On ‘dissolution’ (rapid thawing of the frozen tracer back to room temperature), nuclear polarisation and resulting signal strength decays according to the longitudinal T1 relaxation time of the substrate (~45 s for [1-^13^C]pyruvate). This short period provides the main limitation of the technique. Dissolution back to room temperature must be rapid and involves melting the solid sample with a pressurised superheated liquid, and the metabolic reactions of interest must be fast enough to ensure signal enhancement is not lost before metabolite spectra are recorded.^14^ Speed from dissolution to injection requires polariser integration into the CMR suite with added space for the substrate to undergo a quality control process for clinical application.^16^ Despite these difficulties, automated dissolution has now become a reality, and real-time cardiac metabolic assessment via CMR is possible.^13 17^

## Hyperpolarised MR: implications for the diagnosis and treatment of coronary artery disease

After work in the in vivo rat heart demonstrated that PDH flux and its physiological alterations could be studied non-invasively using [1-^13^C]pyruvate,^18^ animal models of ischaemia have been used to characterise for the first time the in vivo metabolic changes outlined above. In the isolated perfused rat heart after 10 min of global ischemia, residual ischaemia immediately at reperfusion (and delivery of the hyperpolarised pyruvate tracer) is demonstrated by a rise in [1-13C] lactate. Normalisation of PDH flux (and re-appearance of H^13^CO_3_- / ^13^CO_2_) was shown to follow 20 minutes later. Localisation of ischaemia to an arterial territory and quantifying its burden is of clinical interest and has been demonstrated. Raised lactate and reduced H^13^CO_3_^-^ were seen in the anterior wall in an ex-vivo left anterior descending (LAD) infarct model^19^ and in the anterior and anterolateral segments in a left coronary ischaemia reperfusion model,^20^ relative to remote myocardium. After chronic infarction (with in vivo imaging undertaken 4 weeks after in vivo LAD ligation), the complete absence of both metabolites clearly denoted non-viable tissue.^19^

In vivo experience of the technique in a mid-LAD ischaemia reperfusion model in pigs confirmed the potential for human translation. Using a remotely operated balloon occlusion device, hyperpolarised imaging could study metabolic alterations after reperfusion. Pyruvate delivery and hence its metabolism were blunted during a 10 min coronary occlusion with both H^13^CO_3_^−^ and lactate production curtailed in affected segments. The lactate signal rose signifying residual ischaemia immediately on reperfusion, while H^13^CO_3_^−^ remained depressed 5 min after reperfusion.^21^ Similar findings are seen in a circumflex ischaemia reperfusion model.^22^ Ischaemia in such models is a binary event however, and it remains to be seen whether more subtle, lower grade ischaemia, analogous to that (supposedly) encountered during contemporary non-invasive stress testing, can be easily detected.

Myocardial pH falls early and continues to fall in ischaemia due to increased anaerobic glycolysis with associated intracellular proton and lactic acid production.^23^ Complementing the ability to localise the hallmark changes of ischaemic metabolism outlined, hyperpolarised imaging can estimate myocardial pH, potentially rendering its capability to diagnose and localise ischaemia even more sensitive. Mitochondrial CO_2_, produced by PDH, rapidly equilibrates with HCO_3_^−^ due to carbonic anhydrase activity. Measuring the ratio of ^13^CO_2_ and ^13^HCO_3_^−^ and applying the Henderson-Hasselbalch equation allowed an estimation of myocardial pH both before and after ischaemia in the isolated perfused heart subject to 10 min of global ischaemia.^24^ Similarly, the physiological fall in myocardial pH after significant β-adrenergic stimulation was demonstrated in an in vivo model.^25^ Good correlation with ^31^P MRS (which is unable to measure tissue pH in vivo) is lost at pH <6.7 however due to progressive carbonic anhydrase inhibition.^26^

**Figure 3:**
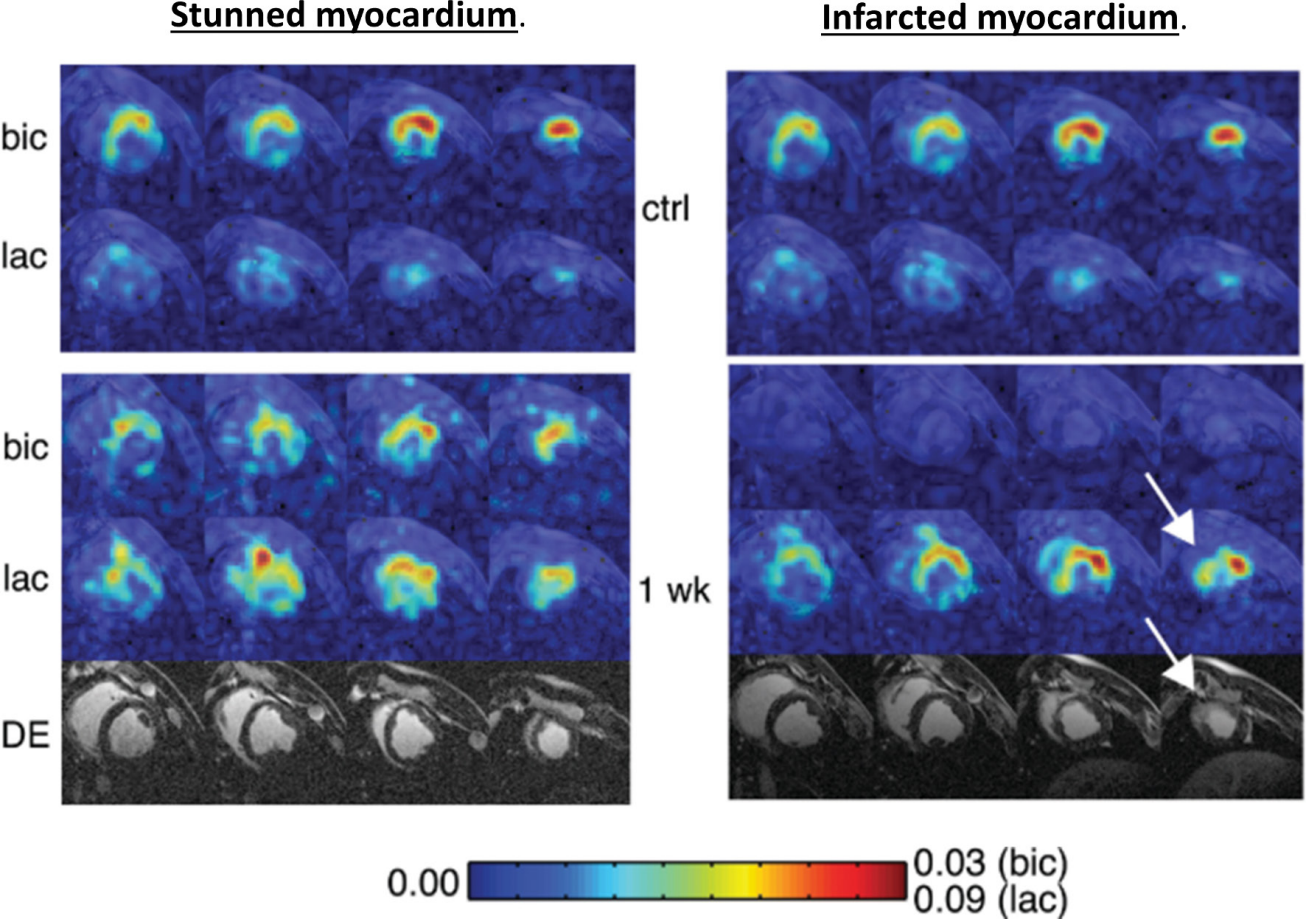
Short axis imaging after 60 min left anterior descending artery (LAD) occlusion in pigs, at baseline and 1 week postreperfusion. Colour intensity is normalised to pyruvate seen in the left ventricle (LV) cavity, stunned myocardium (shown on the left) demonstrated normalisation of HCO_3_^-^, absence of delayed enhancement and normalisation of function at 1 week. In infarcted myocardium (right) HCO_3_^-^ production remained absent at 1 week, lactate was only seen in the peri-infarct region (arrow) and delayed enhancement clearly delineates infarction. (Reproduced with permission *Magn Reson Med* Apr;69:1063–71.

The metabolic downregulation of contractility in response to chronic hypoxia is the hallmark of myocardial hibernation.^27^ The extent of late gadolinium enhancement (LGE) in the index territory informs revascularisation of subtending arteries, with limited enhancement indicating preserved cellular integrity, viable tissue and correlating to recovery of function.^8^ Complementing late enhancement MRI, viability is often assessed by demonstrating contractile reserve during stress echocardiography or tracer uptake during MPS.^28^ Observational data would suggest viability assessed on these grounds was shown to be prognostic in patients undergoing revascularisation.^29^ When prospectively evaluated in the Surgical Treatment for Ischaemic Heart Failure (STICH) trial, however,^30^ any differential survival benefit after surgery (that viability confers) was marginally lost. Hyperpolarised ^13^C pyruvate imaging, by identifying respiring living cells, could complement the current techniques to assess viability and allow us to refine the STICH cohort further such that revascularisation procedures are targeted only to those who potentially stand to benefit. Such power is demonstrated with a 60 min LAD occlusion porcine model of ischaemia, which results in heterogeneous infarcts.^31^ Animals demonstrating transmural non-viable apical infarcts displayed late contrast enhancement during MRI, with myocardium remaining metabolically inactive 1 week postinfarct, denoted by an absence of H^13^CO_3_^−^. In these animals, no restoration of wall motion occurred. Conversely, wall motion returned when HCO_3_^−^ signal normalised after reperfusion, and no late enhancement was seen. Wall motion recovery correlated inversely with the extent of LGE but positively with H^13^CO_3_^−^ production (figure 3).

## Hyperpolarised imaging: understanding the evolution of the metabolic phenotype during the progression to heart failure

^31^P MRS has been instrumental in studying changes in energy homeostasis occurring in heart failure. Of prognostic significance,^32^ the high energy phosphocreatine reserve pool is depleted^33^ with the creatine kinase system failing to maintain ATP levels during the later stages of disease.^34^ Such changes lie downstream of alterations in substrate handling, which before DNP could not be investigated easily using cardiac MR owing to the low natural abundance of (and thus detectable signal from) ^13^C. Heart failure is thus an energy deficient state, analogous to an engine out of fuel.^35^ To satisfy an insatiable metabolic demand, the heart displays versatility in its catabolic substrates, being able to adapt to their varying bioavailability and its own energy requirements. In health, the heart derives the majority of ATP from the β-oxidation of circulating free fatty acids.^36^ The failing heart decreases fatty acid oxidation in preference for more oxygen efficient carbohydrates as a primary fuel source.^37 38^ Beta oxidation is downregulated; glucose uptake and glycolysis increased but PDH flux depressed (likely in part a consequence of the insulin resistance seen in heart failure).^39 40^ Reversing this switch has shown promise as a therapeutic strategy; dichloroacetate (a potent indirect PDH activator) increased stroke volume without increased oxygen uptake when infused in heart failure patients,^41^ and perhexiline (a CPT 1 inhibitor) was shown to increase exercise capacity and left ventricular function.^42^ With PDH regulating the balance between fatty acid and glucose oxidation, hyperpolarised pyruvate imaging can characterise substrate switching and the stepwise metabolic changes seen at different stages in the natural history of disease, with implications for treatment. Using pyruvate, the study of glucose oxidation and the contribution of carbohydrate to the acetyl-CoA pool is measured directly; the contribution of fatty acids to energy production can be inferred. Oxidation of these substrates in health is reciprocally regulated.^43^ The short-chain fatty acid butyrate has shown promise as a hyperpolarised tracer for the direct assessment of fatty acid metabolism; however, with work demonstrating that when combined with [1–^13^C]pyruvate, fatty acid and glucose metabolism can be probed synchronously.^44^

With repeated hyperpolarised scanning at different time intervals, sequential metabolic changes at different stages in the natural history of disease can be studied. This is eloquently demonstrated looking at substrate handling in animal models during progression towards the onset of systolic dysfunction in heart failure. In a physiological pressure overloaded rat model of compensated ventricular hypertrophy using abdominal aortic banding,^45^ PDH flux as assessed by H^13^CO_3_^-^ production did not change during the development of ventricular hypertrophy. ^13^C lactate significantly increased however, indicating a static flux through PDH with increased glycolysis and an uncoupling of glucose metabolism developing in the early compensated hypertrophy process. The spontaneously hypertensive rat model developing severe ventricular hypertrophy seemingly produces contradictory results; both PDH flux and TCA flux (assessed via [2–^13^C]pyruvate) markedly increase, demonstrating a move to favour glucose oxidation.^46^ This inconsistency may be explained by the model having a CD36 deficiency(limiting fatty acid uptake) but may also be related to the model being more aggressive with animals developing much greater degrees of hypertrophy. Therefore, the switch to increased PDH flux and glucose oxidation may well be a later event in the compensatory mechanism, after an initial increase in glycolysis, signifying risk towards imminent systolic dysfunction and decompensation. Finally, synchronous to the onset of mechanical systolic dysfunction comes metabolic decompensation with loss of metabolic flexibility; PDH flux and glucose oxidation become markedly impaired. Such timings were demonstrated in a pacing-induced porcine model of heart failure. H^13^CO_3_^−^ production fell markedly after the DCM phenotype developed, with reduction in Krebs flux (reduced [1-^13^C]glutamate) and impaired cardiac energetics (reduced Phosphocreatine (PCr)/Adenosine triphosphate (ATP)) seen earlier during the later stages of compensation.^47^

The longer term sequential metabolic alterations after myocardial infarction have been studied at different times points after 50 min of LAD territory ischaemia in rat.^48^ Cardiac Krebs cycle flux was found to decrease in a manner correlating to the degree of LV systolic dysfunction only after 6 weeks, with a reduction in PDH flux lagging still further. Glucose oxidation and the pyruvate contribution to energy production, interestingly, clearly lag mechanical dysfunction in the infarcted heart. Mechanisms underlying diabetic cardiomyopathy, characterised by fibrosis, hypertrophy and independent of coronary disease^49^ have been studied extensively using hyperpolarised MR. Here, the heart loses its flexibility to switch to glucose oxidation during elevated workloads, rendering it more susceptible to ischaemic stress.^50^ PDH flux, and thus the potential for glucose oxidation, was shown to be reduced by 80% in diabetic rats.^51^ This is restored, along with diastolic function, after dichloroacetate administration.^52^ Collectively, such models highlight the power of hyperpolarised MR to characterise a changing metabolic phenotype during disease progression, uncovering events occurring independently to the macroscopic changes observed with conventional imaging. Such synergy could inform the planning, choice and timing of treatment of various diseases.

## Hyperpolarised imaging: barriers to clinical human translation

**Figure 4:**
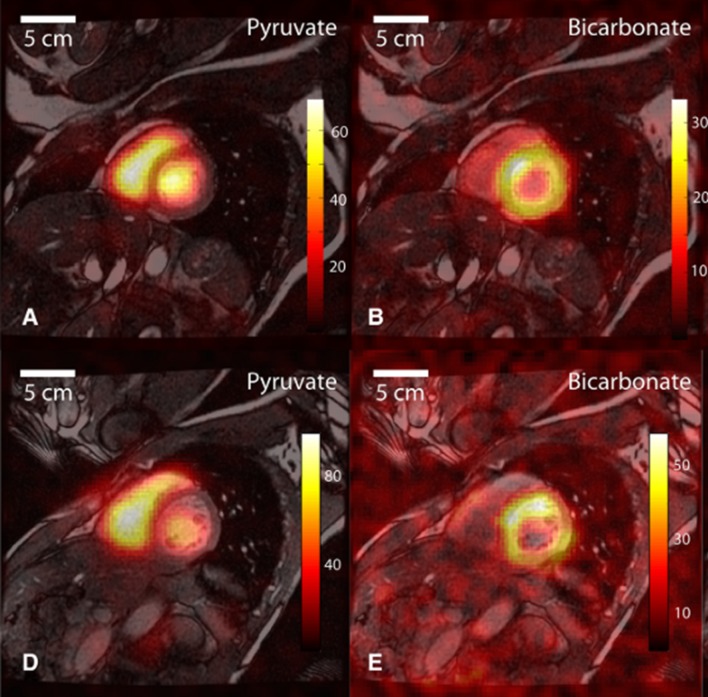
^13^C images in a midventricular slice from two healthy human volunteers (A and B, D and E). [1–^13^C]pyruvate is most clearly seen in the blood pool within the right and left ventricles (A and D). H^13^CO_3_^-^ signal is mostly seen within the LV myocardium. (Reproduced with permission *Circ Res* 2016;119:1177–1182).

Human applications of hyperpolarised MR have already shown clinical benefit. In oncology, the increased reliance on anaerobic metabolism of malignant tissue is widely recognised; [1-^13^C]lactate localisation correctly delineates biopsy proven malignant prostate tissue after [1–^13^C]pyruvate injection.^53^ Investigations of cardiac metabolism have now begun; after [1–^13^C]pyruvate administration to healthy volunteers, H^13^CO_3_^−^ is seen in all ventricular walls (figure 4).^17^ Interpretation of the myocardial lactate signal proved more difficult. An appreciable conversion of the administered [1–^13^C]pyruvate by extracardiac LDH resulted in a diffuse lactate signal distribution encompassing both the blood pool and the cardiac tissue, but techniques to adequately suppress this confounding signal are currently in development.

Current technical barriers to the widespread adoption of hyperpolarised ^13^C technology’s successful application likely include the high cost of the clinical grade hyperpolariser system itself and the need to acquire additional ^13^C capable MR hardware (^13^C coils, amplifiers and so on), which many commercially available scanners are not equipped with as standard. Such additional requirements make DNP more than a simple adjunct to standard proton MR imaging. The technique to date has largely been demonstrated in the preclinical setting; the spatial and temporal resolutions achieved in such studies may not be representative of the gradient capabilities achievable by typical clinical scanners. Given a limited non-recoverable amount of ^13^C signal, there is a trade-off between temporal and spatial resolution while maintaining an acceptable signal to noise (SNR) ratio. Generating temporally resolved kinetic data is not feasible with current technology if a clinically useful spatially resolved image is desired. An assumption is thus required that the time point of imaging is that encompassing the maximum signal for the metabolite of interest. Current preclinical techniques, however, *can* generate spatially resolved imaging, using summed spectral data over time to prevent such an error. The suitability of a compound for use as a hyperpolarised tracer is limited by the T1 relaxation time, and fast cellular uptake and metabolism are prerequisites if we are to expect appreciable signals of metabolites before the signal enhancement decays away. Such drawbacks limit the number of metabolic reactions that can be studied. Because of the typically short T1 relaxation times, speed is essential to minimise signal decay; dissolution from 1K, a quality control process, and transfer to a power injector, must happen in a matter of seconds, which represents a technical challenge. The signal is non-recoverable, and the sample takes up to 3 hours to polarise meaning with each patient prepared for imaging, there is little room for error. Active research to address these limitations is underway.

Despite these difficulties, this novel technology will extend our understanding of in vivo metabolism, and how it changes in response to alterations in workload, energy substrate availability and, finally, pathology. Such metabolic profiling may complement the current array of non-invasive cardiac imaging to ultimately benefit patients. Finally, directly diagnosing ischaemia, defining viability and identifying metabolic precursors to heart failure in at risk populations will allow us to refine the practice of invasive cardiology, and target the hugely expensive pharmacotherapy of tomorrow, to the patients that stand to benefit the most.
